# Gene Expression Profiling of Classically Activated Macrophages in *Leishmania infantum* Infection: Response to Metabolic Pre-Stimulus with Itaconic Acid

**DOI:** 10.3390/tropicalmed8050264

**Published:** 2023-05-03

**Authors:** Génesis Palacios, Elva Vega-García, Basilio Valladares, José Antonio Pérez, Roberto Dorta-Guerra, Emma Carmelo

**Affiliations:** 1 Instituto Universitario de Enfermedades Tropicales y Salud Pública de Canarias (IUESTPC), Universidad de La Laguna (ULL), Avenida Astrofísico Francisco Sánchez s/n, 38200 La Laguna, Tenerife, Spain; gpalacio@ull.edu.es (G.P.);; 2 Departamento de Obstetricia y Ginecología, Pediatría, Medicina Preventiva y Salud PÚblica, Toxicología, Medicina Legal y Forense y Parasitología, Universidad de La Laguna, 38200 La Laguna, Tenerife, Spain; 3 Departamento de Bioquímica, Microbiología, Biología Celular y Genética, Facultad de Ciencias, Universidad de La Laguna, 38200 La Laguna, Tenerife, Spain; 4 Departamento de Matemáticas, Estadística e Investigación Operativa, Facultad de Ciencias, Universidad de La Laguna, 38200 La Laguna, Tenerife, Spain

**Keywords:** gene expression profiling, macrophage activation, classically activated macrophages, metabolic stimulus, itaconate, *Leishmania infantum*

## Abstract

*Leishmania* infection of phagocytic cells, such as macrophages, induces the differentiation of infected cells into different phenotypes according to their surrounding microenvironments. The classical activation of macrophages involves metabolic reprogramming, in which several metabolites such as succinate, fumarate and itaconate are accumulated. The immunoregulatory functions of itaconate in the context of *Leishmania* infection were investigated in this paper. Ex vivo bone marrow-derived macrophages were differentiated into classically activated macrophages through IFNG activation and infection with *Leishmania infantum*. A high-throughput real-time qPCR experiment was designed for the analyses of 223 genes involved in immune response and metabolism. The transcriptional profile of classically activated macrophages revealed the enrichment of the IFNG response pathways and the upregulation of genes such as *Cxcl9*, *Irf1*, *Acod1*, *Il12b*, *Il12rb1*, *Nos2* or *Stat1*. In vitro pre-stimulation with itaconate induced a loss of the parasite control and the upregulation of genes related to local acute inflammatory response. Our results reveal that itaconate accumulation dampened classically activated macrophage antiparasitic activity, and this is reflected by the differential expression of the *Il12b*, *Icosl* and *Mki67* genes. The possibility of inducing parasite-killing responses in the host through metabolic reprograming is an interesting approach for the treatment of *Leishmania* infections that will undoubtedly attract increasing attention in the coming years.

## 1. Introduction

The term leishmaniasis corresponds to a spectrum of diseases presenting different forms, from self-healing to fatal, caused by several species of the genus *Leishmania* [1]. Visceral leishmaniasis (VL) is the most severe form of the disease and is caused by *L. donovani* or *L. infantum* [2], affecting mainly the spleen, liver and bone marrow of vertebrate hosts. During *L. infantum* infection, macrophages have a key role as the main cellular target.

Macrophages are professional innate immune cells involved in development, homeostasis, tissue repair and immunity [3,4]. They shift their functional state according to the surrounding microenvironments and stimuli. The terms “activation” or “polarization” of macrophages refer to the ability of naïve macrophages (M0) to respond to environmental stimuli, such as cytokines or Toll-like receptor (TLR) agonists, and, subsequently, induce distinct patterns of gene and protein expression that change their phenotype and physiology [5]. This activation process has been traditionally classified into “classical” or “alternative” activation, depending on the stimulus, generally IFNG or IL4, respectively [6,7]. M1 are considered inflammatory macrophages (producing a low amount of IL10) and M2 are involved in anti-inflammatory mechanisms (producing a low to a high amount of IL10) [4].

M1 polarization requires two signals: the first is a “priming” signal induced by IFNG, which is produced by natural killer (NK) cells and Th1 cells, and the second signal is produced by a Toll-like receptor (TLR) ligand, such as LPS [8]. Macrophage polarization involves metabolic reprogramming; therefore, depending on the stimuli, they can switch from an aerobic profile, based on oxidative phosphorylation, to an anaerobic one, based on glycolysis, and vice versa [9]. Upon classical macrophage activation, an initial induction of the Krebs cycle and oxidative phosphorylation (OXPHOS) has been described, followed by nitric oxide-mediated disruption of the Krebs cycle and the respiratory chain [10], resulting in the accumulation of succinate, fumarate and itaconate, which exhibit a wide range of immunoregulatory functions [11,12,13,14,15]. Itaconate is derived from cis-aconitate by the enzyme cis-aconitate decarboxylase (ACOD1), encoded by *Acod1*, also known as immunoresponsive gene 1 (*Irg1*). In this study, we aimed to explore macrophage polarization by mimicking the in vitro M1 polarization state that happens in *Leishmania infantum* infection. During infection, inoculated *L. infantum* promastigotes are taken up by phagocytic cells, including macrophages and neutrophils. In macrophages, parasites are internalized into the phagolysosome and differentiated into amastigotes in the parasitophorous vacuole, where parasites can persist [16,17].

The *Irg1* gene has been described to be upregulated in tissue infected with *L. infantum* [18,19]. Considering that itaconate and *Irg1* are upregulated during macrophage activation, we investigated the consequences of exogenous itaconate exposure prior to macrophage activation in *Leishmania infantum* infection: if *ACOD1* catalyzes the upregulation of itaconate, how would an excess of itaconate affect the infection process?

The expression of some M1 markers at different timepoints during mouse liver infection by *L. infantum* has been described [18], but a large-scale transcriptional profile of the activation process of infected macrophages remains unexplored. For this purpose, murine bone marrow-derived macrophages (BMDMs) were differentiated ex vivo and subjected to IFNG stimulus and infection by *L. infantum* to obtain classically activated macrophages. In addition, other experimental conditions were included, particularly pre-stimulation with itaconic acid to determine the effect of this metabolite on macrophage activation.

In our study, high-throughput real-time qPCR was used as a platform for the analysis of 223 genes related to immune response and metabolism. This technology allows the precise correction of experimental variations through normalization and also avoids the need for validation using qPCR, as in other methodologies (RNA-seq, microarrays, etc.) [18,20,21]. Differentially expressed genes and multivariate analysis allowed us to identify the most significant changes in each condition evaluated.

In our experiment, after infection, the parasitic burden on classically activated macrophages was reduced by half, and the upregulation of genes involved in the innate and interferon gamma responses was observed. Itaconic acid pre-stimulus in classically activated macrophages also upregulated these genes, but the parasite load was the same as that observed in control cells. *Il12b* gene expression is crucial in this process of macrophage activation, but its overexpression drops upon itaconic acid pre-stimulation. Itaconic acid pre-stimulus in the absence of IFNG activation induced the upregulation of genes of the local acute inflammatory response (*Il6* and *Cxcl9*), which were related to the increased parasite load. On the other hand, the effect of itaconic acid pre-stimulus on activated macrophages was mediated by the downregulation of *Il12b* and upregulation of *Icosl* and *Mki67*.

The possibility of activating antiparasitic responses by immunometabolic reprogramming, as explored in this paper, is tempting, given the increasing evidence on the interconnection between macrophage immune responses in *Leishmania*-infected cells and metabolic pathways such the Krebs cycle or the respiratory chain [17,22,23]. Our large-scale transcriptional analysis of the intermediates involved in the immune and metabolic pathways in macrophages contributes to the identification of the molecular mechanisms underlying infection, which may be useful in the search for immunometabolic strategies for infection control.

## 2. Materials and Methods

### 2.1. Collection and Culture of Murine Bone Marrow-Derived Macrophages (BMDM)

The collection and culture of BMDM were performed with RPMI medium (Gibco BRL, Grand Island, NY, USA) supplemented with 2 mM L-glutamine, 10% inactivated fetal calf serum (SFBI), 2 g/L CO_3_NaH and 100 U/mL of Penicillin streptomycin (Gibco™), hereafter referred to as RPMI medium for macrophages. All procedures were performed under sterile conditions.

Three mice (8-week-old males) were euthanized using standard procedures, and their femurs were removed. The collection of BMDM was performed as described in a reported protocol [4], but with some modifications. Femurs were sterilized for 10–20 s with 70% ethanol and washed with PBS in Petri dishes. Flushing was performed with RPMI medium for macrophages, and after the centrifugation of the cell suspension, the pellet was resuspended in 1× RBC Lysis buffer (eBioscience™, Invitrogen, Carlsbad, CA, USA) for 1 min. Cells were washed twice with RPMI medium, cell viability was checked and a cell count was performed to seed 1 × 10^6^ cells/well in 6-well cell culture plates (Gibco, Life Technologies, Paisley, UK) in RPMI medium for macrophages supplemented with 20 ng/mL of M-CSF (ProSci, Poway, CA, USA). Plates were incubated for 3 days at 37 °C + 5% CO^2^. After that, half of the medium was added to each well to induce macrophage differentiation, and plates were incubated for 4 more days. At day 7, cell viability was checked by using trypan blue exclusion, and BMDM cell monolayers were washed with PBS.

### 2.2. Stimulation with IFNG and Itaconic Acid

Each experimental condition was assayed in triplicate. BMDM monolayers, in respective groups (Table 1), were exposed to itaconic acid at 7.5 mM [24]. Cells were incubated for 3 h and washed with PBS. Subsequently, recombinant mouse IFNG (50 ng/mL) was added to RPMI (with 10% heat-inactivated fetal bovine serum (FBS)) and incubated for 12 h.

### 2.3. BMDM Infection with Leishmania Infantum

Macrophages were infected with promastigotes of *L. infantum* (JPC strain, MCAN/ES/98/LLM-724) in the stationary growth phase. Previously, the promastigotes were cultured in RPMI medium (Gibco BRL, Grand Island, NY, USA) supplemented with 20% FBS, 100 μg/mL streptomycin (Sigma-Aldrich, St. Louis, MO, USA) and 100 U/mL of penicillin (Biochrom AG, Berlin, Germany) at 26 °C until they reached the stationary phase.

Parasites were washed with PBS and resuspended in RPMI + 10% SFBI (medium for macrophages), and 2 mL were added to each well. Infection was performed in a 1:10 ratio (macrophages: *L. infantum*), and they were incubated for 24 h. Thereafter, cell viability was checked, and wells were washed with PBS to remove non-internalized parasites. RPMI medium for macrophages was added, and cells were incubated for 24 h. All experiments were performed in triplicate.

### 2.4. DNA and RNA Isolation

DNA and RNA were obtained from BMDM culture samples using an All prep DNA/RNA mini kit (Qiagen, Hilde, Germany), following the manufacturer’s instructions. Nucleic acid quantification was determined as previously described [18]. RNA integrity number (RIN) was >7.3 for all RNA samples included in this study.

### 2.5. Real-Time qPCR for the Evaluation of Parasite Load

Relative quantification was used for the evaluation of parasite load in infected macrophages, using the 18S rRNA gene of *Leishmania* sp. as the target gene [25,26,27,28,29] and *Hprt* of *Mus musculus* as the reference gene. For the amplification of the 18S rRNA gene of *Leishmania* sp., a primer reported in the literature [29,30] was used, 18S-R223 (TCCCATCGCAACCTCGGTT), and the reverse primer 18SR (AAAGCGGGCGCGGGCGGTGCTG) was designed to obtain an amplicon with a length of 350 bp. For the amplification of the *Hprt* gene, a primer pair was designed: forward primer HPRT1F (ATGTCATGAAGGAGGAGATGGGA) and reverse primer HPRT1R (ATCCAGCAGCAGGTCAGCAAAGA). In this case, the size of the amplicon obtained was 87 bp.

The qPCR reactions were prepared in a final volume of 20 µL, with 10 µL of 2× qPCRBIO SyGreen Mix master mix (PCR Biosystems, London, UK), 1 µL of each primer (at a concentration of 0.2 µM) and 5 µL of sample (5 ng of DNA per reaction). Thermal profiling consisted of a first cycle at 95 °C for 2 min, followed by 40 cycles of amplification at 95 °C for 5 s, 60 °C for 20 s and 72 °C for 30 s. Final elongation was performed at 72 °C for 1 min. The qPCR reactions were run in a thermal cycler LightCycler^®^ 480 (Roche, Basel, Switzerland). Cq (quantitative cycle) values were used for relative quantitative data analysis using the Pfaffl method [31], using the parasitic load of untreated infected macrophages as a reference.

### 2.6. Reverse Transcription and High-Throughput Real-Time Quantitative PCR

The reverse transcription process was conducted using a High Capacity cDNA Reverse Transcription kit (Thermo Fisher, Waltham, MA, USA) and Veriti^®^ 96-Well Fast Thermal Cycler thermocycler (Thermo Fisher) as previously described [18]. OpenArray^®^ plates with TaqMan probes (Thermo Fisher) were utilized for real-time qPCR to amplify 223 genes associated with various processes such as innate and adaptive immune response, lipid metabolism, prostaglandin synthesis, the MAPK signaling pathway and C-type lectin receptors (Table S1). The QuantStudio^TM^ 12K Flex Real-Time PCR System (Thermo Fisher Scientific) was used for thermal cycling and fluorescence detection following the manufacturer’s guidelines, with triplicate amplification for each sample. Cq values produced by this platform are already adjusted for amplification efficiency [20,21].

### 2.7. Data Pre-Processing and Processing

Data were exported from QuantStudio^TM^ 12K Flex Real-Time PCR System software v.1.2.2 to MS Excel. The arithmetic average quantitative cycle (Cq) was used for data analysis. *GenEx* (MultiD) software was used for data preprocessing and normalization. Reference genes for this dataset were selected with *GenEx* using two different methods: geNorm (setting M-value lower than 0.5) and NormFinder. Two genes that showed the most stable expression, *Ubc* and *Stat6*, were used to calculate normalized relative quantity (NRQ) values. 

### 2.8. Representations of Differential Gene Expression

Differentially expressed genes (DEGs) between biological groups analyzed (i.e., infected mice) and control mice at each timepoint are represented in volcano plots. The fold change (FC) of gene expression was calculated as the ratio between the average gene expression in the infected or treated group and control group and is expressed as Log_2_. The criteria to consider differentially expressed genes (DEGs) were the statistical significance threshold of *p*-value ≤ 0.05 and the biological significance threshold of log_2_FC >1 or log_2_FC <−1 (corresponding to FC >2 and FC <0.5, respectively).

In the volcano plots, genes above the biological significance and statistical significance thresholds are represented in red and blue, referring to their upregulation and downregulation, respectively. Volcano plots were drawn by plotting the −log_10_ *p*-value (determined by using the two-tailed Mann–Whitney U test) on the y-axis and the log_2_ of FC on the x-axis. These graphics were generated using SPSS version 26 (IBM Corporation, Armonk, NY, USA) statistical software.

### 2.9. Statistical Analysis

For each parameter evaluated, the mean and standard error (SEM) were computed. Outliers were scrutinized through an inspection of boxplots and studentized residuals, and the normal distribution of data was verified with either the Kolmogorov–Smirnoff test or the Shapiro–Wilk test, as appropriate. Additionally, the Levene test was employed to check the homogeneity of variance.

The dataset was examined using principal component analysis (PCA) of NRQ values to determine its underlying structure. In assessing the correlation between variables, the correlation matrix was inspected. The Kaiser–Meyer–Olkin (KMO) measure and Bartlett’s test of sphericity were examined. These assessments implied that the data were likely to be factorizable. Plot representations were obtained in SPSS 26 (SPSS Inc., Chicago, IL, USA). Differences in scores of plotted principal components between the groups were confirmed by one-way ANOVA followed by Tukey’s post hoc tests or by a two-tailed unpaired *t*-test with data with normal distribution, using GraphPad Prism version 9.2.0, GraphPad Software, San Diego, CA, USA.

NRQ values were also used for individual gene representation and representations of differential gene expression. Statistical differences in NRQ values were analyzed by using a one-way two-tailed unpaired *t*-test or two-tailed Mann–Whitney U test, as appropriate, using SPSS version 26 (IBM Corporation, Armonk, NY, USA) statistical software.

### 2.10. Enrichment Analysis

To compare the biological processes and pathways over-represented in selected groups, gene set enrichment analysis (GSEA) was performed with NRQ values of all genes in the dataset, using GSEA software (version 4.1.0, San Diego, CA, USA). The Metric for ranking genes was the tTest ratio. Each pathway attained a normalization enrichment score (NES). Enrichment analysis was performed using the “Mouse_GOBP_AllPathways_no_GO_iea”, “Mouse_Human_MSigdb” or “Mouse_Human_Reactome” gene sets, as appropriate [32]. The leading edge analysis was performed in GSEA with the following criteria: *p*-value < 0.05 and false discovery rate (FDR) < 0.25.

## 3. Results

In order to analyze the response of activated macrophages to *Leishmania infantum* infection under the effect of a metabolic pre-stimulus such as itaconic acid, five different biological groups were assembled using mice macrophages: non-activated and uninfected macrophages (M0), infected macrophages (Li), classically activated macrophages (exposed to IFNG and infected with *L. infantum*, group M1), non-activated macrophages pre-stimulated with itaconic acid (Li-Ita) and classically activated macrophages pre-stimulated with itaconic acid (M1-Ita) (Table 1). Differences in parasite load under the different stimuli were determined using qPCR as relative to infected macrophages (Li). A large-scale transcriptional profile was obtained for each group. Gene expression results were analyzed using principal component analysis (PCA) and compared using the fold change expression to determine the genes responsible for the differences between the biological groups. Additionally, gene set enrichment analysis (GSEA) was performed to determine the pathways and processes involved in each condition.

### 3.1. Itaconic Acid Abrogates the Control of Parasite Replication in M1 or Classically Activated Macrophages Infected with L. infantum

**Figure 1:**
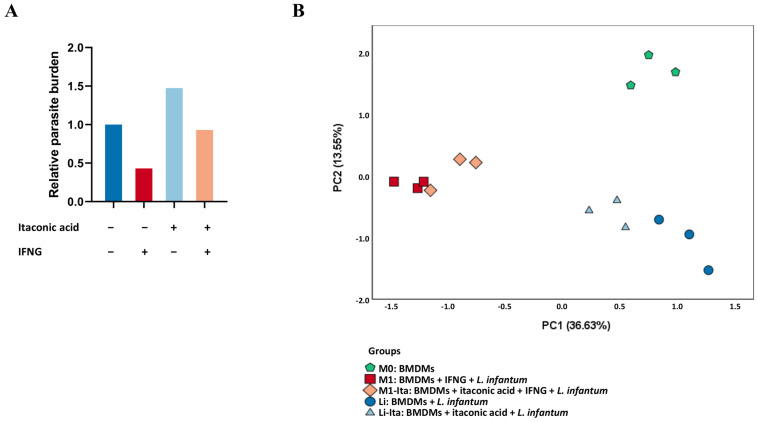
(**A**) Relative parasite burden in mouse macrophages infected in vitro with *L. infantum* and stimulated or not with IFNG for 12 h, with or without pre-stimulus with itaconic acid for 3 h. Dark blue bar represents group Li; red bar: group M1; light blue bar: group Li-Ita; orange bar: group M1-Ita. The results represent the average of three experiments. Parameters to obtain the relative parasite burden are indicated in Materials and Methods. Fold change compared to control group (Li: infected macrophages). (**B**) Principal component analysis of NRQ (normalized relative quantity) values of gene expression of 223 analyzed genes in all groups: non-activated and uninfected macrophages (green pentagons), infected macrophages (blue circles), non-activated macrophages pre-stimulated with itaconic acid (light blue triangles), classically activated macrophages (red squares) and classically activated macrophages pre-stimulated with itaconic acid (orange squares). Principal components 1 (PC1) and 2 (PC2) are plotted on the x- and y-axis, respectively, with the proportion of total variance related to each principal component (PC) indicated.

The activation of macrophages infected with *L. infantum* (M1) with IFNG caused a reduction in the parasite burden by half compared to non-activated macrophages (Figure 1A). In contrast, when non-activated macrophages were pre-stimulated with itaconic acid (Li-Ita), the parasite burden increased by 50% compared to non-stimulated cells. Surprisingly, the relative parasite load in classically activated macrophages pre-stimulated with itaconic acid (M1-Ita) was similar to that of non-activated infected macrophages.

Despite this similarity, gene expression profiles in these two groups were very different, as revealed by using PCA (Figure 1B). The structure of the dataset was determined, and three principal components were extracted, explaining 60% of the total variance. Principal component 1 (PC1) accounted for 36.6% of variance, and it was the main driver of the differences observed between IFNG-activated macrophages (red and orange in Figure 1B) and all other experimental groups (Figure 1B). Non-activated and uninfected macrophages (M0, green pentagons in Figure 1B) and infected macrophages (Li, deep blue circles) showed statistically significant differences in the score of PC1 compared to activated macrophages (M1, red squares; *p* < 0.0001) and compared to activated macrophages pre-stimulated with itaconic acid (M1-Ita, orange diamonds; *p* < 0.0001). Thus, activated macrophages are separated from all other data points through principal component 1 (PC1). Similarly, non-activated macrophages pre-stimulated with itaconic acid (Li-Ita) displayed a particular gene expression profile, showing statistically significant differences in the score of PC1 compared to infected macrophages (Li; *p* = 0.0104), in comparison to activated macrophages (group M1; *p* < 0.0001) and also compared to classically activated macrophages pre-stimulated with itaconic acid (M1-Ita; *p* < 0.0001).

In Figure 1B, non-activated and uninfected macrophages (M0) showed statistically significant differences in the score of PC2 compared to all other groups (*p* = 0.0002). That is, infected macrophages are separated from other data points due to principal component 2. Additionally, infected macrophages (Li) showed statistically significant differences in the score of PC2 compared to activated macrophages (M1; *p* = 0.0120) and compared to activated macrophages pre-stimulated with itaconic acid (M1-Ita; *p* = 0.0030).

Therefore, Figure 1A reveals that pre-stimulus with itaconic acid abrogates the control of parasitic burden in activated macrophages, but also in non-activated macrophages. In addition, Figure 1B shows that classically activated macrophages displayed a particular transcriptional profile (irrespective of itaconic acid pre-stimulus), different from non-infected and non-activated macrophages. In contrast, despite showing very similar parasite load, groups Li (infected) and M1-Ita (infected, activated, pre-stimulated with itaconic acid) exhibited very different transcriptional profiles, as revealed by the statistically significant differences in both PC1 and PC2. At this point, our goal was to determine the mechanisms (in terms of transcriptional profile changes) whereby itaconate stimulation increases parasite load in non-activated macrophages and also abrogates the observed reduction in parasite load due to macrophage activation with IFNG.

### 3.2. M1 or Classically Activated Macrophages Infected with L. infantum Control Parasite Replication through Upregulation of Genes Involved in Innate and Defense Response

**Figure 2:**
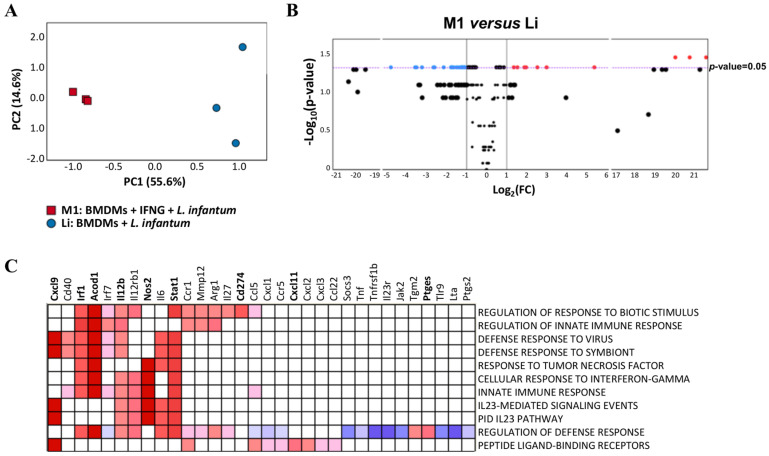
(**A**) Principal component analysis of NRQ values of 223 analyzed genes in infected macrophages (blue circles) and classically activated macrophages (red squares). Principal components 1 (PC1) and 2 (PC2) are plotted on the x- and y-axis, respectively, with the proportion of total variance related to each principal component (PC) indicated. (**B**) Volcano plot with the differential expression of 223 analyzed genes in classically activated macrophages (M1) vs. infected macrophages (Li). The results represent the average of three experiments. Biological threshold is indicated by black vertical lines. Statistical threshold (*p*-value = 0.05) is indicated as a purple dotted horizontal line. Blue and red dots represent DEGs downregulated and upregulated, respectively. Black dots are genes without significant statistical differences. (**C**) Core enrichment of significant gene sets in M1. The leading edge analysis was performed in GSEA with the gene sets enriched (with *p*-value < 0.05 and FDR < 0.25). The heatmap shows the clustered genes in the leading edge subsets. Score values in the ranked gene list generated in GSEA are represented as colors, where the range of colors (red, pink, light blue, blue) shows the range of score values (high, moderate, low, lowest). DEGs are in bold.

The classical activation of macrophages by IFNG before infection with *L. infantum* induced extensive changes in gene expression profiles compared to infected non-primed cells (Figure 2). Activated macrophages (M1) were differentiated from non-activated infected macrophages (Li) through PCA (Figure 2A), showing statistically significant differences in the score of PC1 compared to control (Li; *p*-value < 0.0001) due to the expression of genes correlated to PC1, listed in Table S2. Additionally, some of these genes were differentially expressed (colored dots in volcano plot in Figure 2B). Among the DEGs, *Arg1*, *Il6*, *Nos2*, *Acod1*, *Stat1*, *Irf1*, *Ptges* and *Il2rg* were upregulated and *Mki67*, *Cd28*, *Tfgb2*, *Cd80*, *Alox5*, *Ptgs1*, *Il1rn*, *Mapk3*, *Il16* and *Il1b* showed downregulation (Figure 2B).

**Figure 3:**
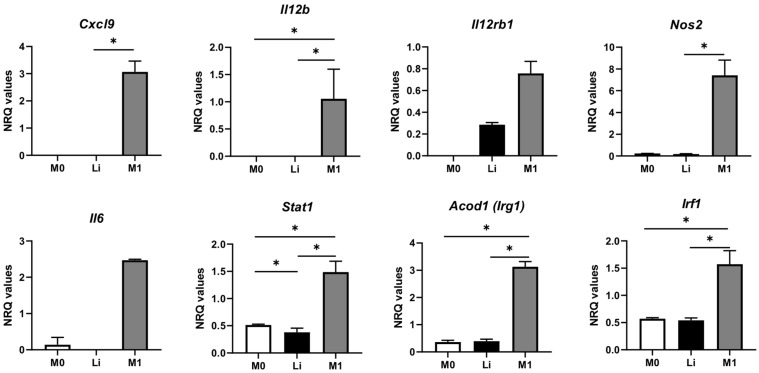
Expression levels of genes with the highest factor loading that explained the expression profile in activated macrophages. These genes were involved in some of the significantly enriched gene sets in M1. Mean normalized relative quantities (NRQ) in M0 (non-activated and uninfected macrophages), Li (infected macrophages) and M1 (classically activated macrophages). The results represent the average of three experiments. Statistically significant differences determined using two-tailed Mann–Whitney test are indicated (* *p* < 0.05).

Functional enrichment using GSEA showed at least 11 annotations enriched in activated infected macrophages (M1) vs. group Li (Figure 2C). Most of the enriched pathways are directly related to innate defense responses to different challenges, such as biotic stimulus or viruses. In particular, *response to tumor necrosis factor* (NES = 1.63; FDR = 0.24), *cellular response to interferon-gamma* (NES = 1.73; FDR = 0.200) and *innate immune response* (NES = 1.61; FDR = 0.23) were strongly enriched in this analysis. Leading edge analysis showed that most of the genes contributing to the enrichment (*Cxcl9*, *Irf1*, *Acod1*, *Il12b*, *Nos2*, *Stat1*, *Cd274*, *Cxcl11* and *Ptges*, indicated in bold in Figure 2C) were also DEGs in M1 (Figure 2B), demonstrating the relevance of all these genes in the activation of macrophages as a response to *L. infantum* infection. These classically activated macrophages (M1) showed upregulated genes such as *Cxcl9*, *Irf1*, *Acod1*, *Il12b*, *Il12rb1*, *Nos2*, *Stat1* and *Il6* (Figure 3).

### 3.3. Itaconic Acid Pre-Stimulus Induces Upregulation of Genes of Local Acute Inflammatory Response in Macrophages Infected with L. infantum

**Figure 4:**
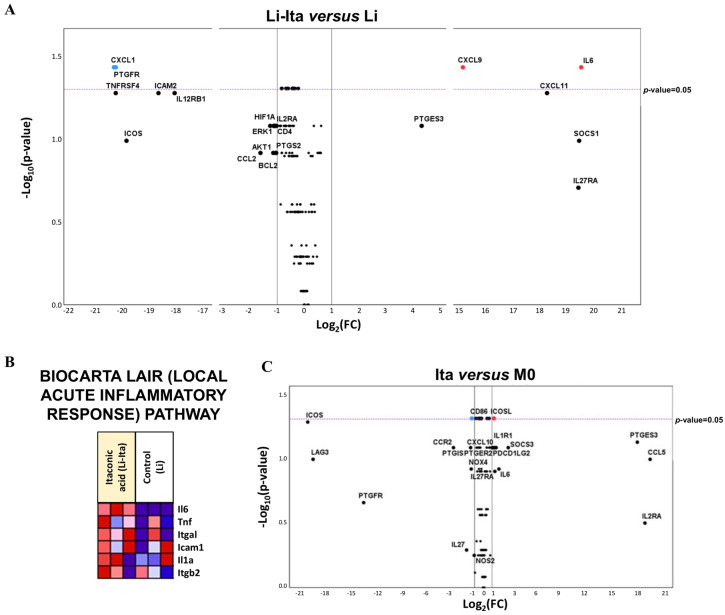
(**A**) Volcano plot with the differential gene expression of 223 analyzed genes in non-activated macrophages pre-stimulated with itaconic acid (Li-Ita) vs. infected macrophages (Li). Biological threshold is indicated by black vertical lines. Statistical threshold (*p*-value = 0.05) is indicated as a purple dotted horizontal line. Blue and red dots represent DEGs downregulated and upregulated, respectively. Black dots are genes without significant statistical differences. (**B**) Biocarta LAIR (local acute inflammatory response) pathway heatmap. This gene set was upregulated in infected macrophages pre-stimulated with itaconic acid. NRQ values are represented as colors, where the range of colors (red, pink, light blue, dark blue) shows the range of expression values (high, moderate, low, lowest). (**C**) Volcano plot with the differential gene expression of 223 analyzed genes in non-activated and uninfected macrophages pre-stimulated with itaconic acid (Ita) vs. non-activated and uninfected macrophages (M0). Blue and red dots represent DEGs downregulated and upregulated, respectively. Black dots are genes without significant statistical differences. Results represent the average of three experiments.

As observed in Figure 1A, itaconic acid pre-stimulus (Li-Ita) caused an increase in parasite burden compared to infected non-activated macrophages (Li); in this comparison, only a statistically significant upregulation of *Il6* and *Cxcl9* and downregulation of *Cxcl1* were observed in the volcano plot (Figure 4A). After performing gene set enrichment analysis (GSEA), the only pathway that was significantly enriched in macrophages infected and pre-stimulated with itaconic acid was the BIOCARTA_LAIR_PATHWAY (NES = 2.04; FDR = 0.059), which is related to local acute inflammatory response (Figure 4B). Remarkably, *Il6* was upregulated, and contributed significantly to the enrichment of this pathway, as seen in Figure 4A,B. Thus, it can be concluded that acute inflammatory response activated by *Il6*, *Tnf*, *Itgal*, *Icam1*, *Il1a* and *Itgb2*, in the context of absence of other immune cells, promotes parasite replication in infected non-activated macrophages. Intriguingly, the effect of itaconic acid pre-stimulus on macrophages, in the absence of infection or IFNG activation, was upregulation of *Icosl* and downregulation of *Cd86*, with statistically significant differences compared to the control (Figure 4C).

### 3.4. Gene Expression Changes Associated with the Effect of Pre-Stimulus with Itaconic Acid in Classically Activated Macrophages in L. infantum Infection

**Figure 5:**
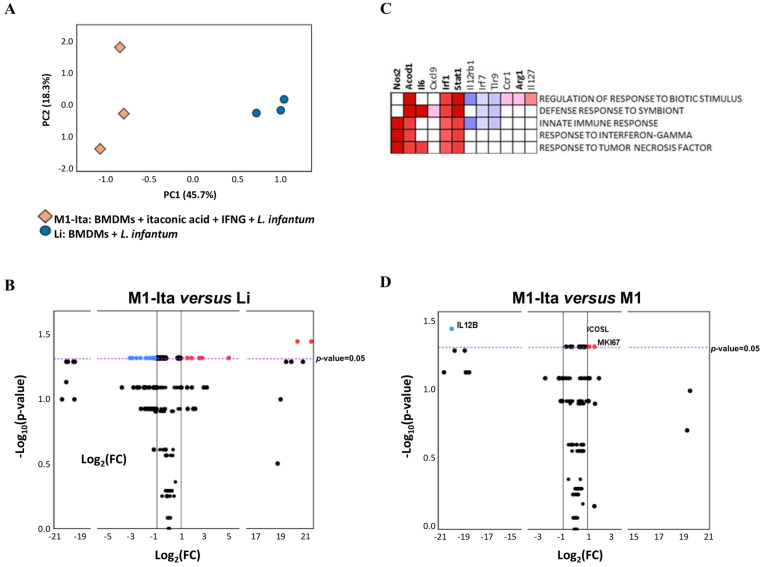
(**A**) Principal component analysis of NRQ values of gene expression of 223 analyzed genes in infected macrophages (blue circles) and classically activated macrophages pre-stimulated with itaconic acid (orange squares). Principal components 1 (PC1) and 2 (PC2) are plotted on the x- and y-axis, respectively, with the proportion of total variance related to each principal component (PC) indicated. (**B**) Volcano plot with the differential expression of 223 analyzed genes in classically activated macrophages pre-stimulated with itaconic acid (M1-Ita) vs. infected macrophages (Li). Biological threshold is indicated by black vertical lines. Statistical threshold (*p*-value = 0.05) is indicated as a purple dotted horizontal line. Blue and red dots represent DEGs downregulated and upregulated, respectively. Black dots are genes without significant statistical differences. (**C**) Core enrichment of significant gene sets in group M1-Ita. The leading edge analysis was performed in GSEA with the gene sets enriched (with *p*-value < 0.05 and FDR < 0.25). The heatmap shows the clustered genes in the leading edge subsets. Score values in the ranked gene list generated in GSEA are represented as colors, where the range of colors (red, pink, light blue, dark blue) shows the range of score values (high, moderate, low, lowest). DEGs are in bold. (**D**) Volcano plot with the differential expression of 223 analyzed genes in classically activated macrophages pre-stimulated with itaconic acid (M1-Ita) vs. classically activated macrophages (M1). Biological threshold is indicated by black vertical lines. Statistical threshold (*p*-value = 0.05) is indicated as a purple dotted horizontal line. Blue and red dots represent DEGs downregulated and upregulated, respectively. Black dots are genes without significant statistical differences. The results represent the average of three experiments.

As described in Figure 1, the parasite load in activated macrophages pre-stimulated with itaconic acid (M1-Ita) is very similar to that observed in group Li, which are non-activated cells, although their expression profile is very different (Figure 5A,B). The volcano plot revealed the differential expression of a large number of genes between these groups: *Cxcl9*, *Cxcl11*, *Il12b*, *Nos2*, *Acod1*, *Il18bp*, *Stat1*, *Cd274*, *Irf1* and *Ptges* showed upregulation and *Mki67*, *Cd28*, *Alox5*, *Tgfb2*, *Ccl9*, *Cd4*, *Mpak3*, *Cd68*, *Cxcr4*, *Hif1a*, *Ifngr2*, *Il16*, *Il1b*, *Tlr2*, *Il6ra*, *P2rx7*, *Rxra*, *Hpgds*, *Mmp14*, *Lta4h*, *Il1rn*, *Tgfbr2* and *Il6st* were downregulated, and all these genes showed statistically significant differences compared to group Li (Figure 5B).

Pathways enriched in M1-Ita vs. Li are indicated in Figure 5C. Remarkably, most of the genes contributing to the enrichment were upregulated: *Nos2*, *Acod1*, *Il6*, *Cxcl9*, *Irf1*, *Stat1* and *Il27*, and were also upregulated and contributing to the enrichment observed in activated macrophages (Figure 2C and Figure 3). Furthermore, some but not all the pathways in the enrichment of M1-Ita vs. Li and M1 vs. Li were the same: *innate immune response*, *response to interferon gamma and response to tumor necrosis factor.* This finding suggests that itaconic acid waives the protective role of IFNG, because it limits the upregulation of the defense pathways observed in classically activated macrophages.

Finally, the effect of itaconate pre-stimulus on infected and activated macrophages (M1-Ita vs. M1) was described in terms of differential gene expression profile. As observed in Figure 1, itaconic acid not only caused increased parasite burden in macrophages infected with *L. infantum*, but also abrogated the reduction in parasite load that occurs in macrophages due to activation with IFNG. Figure 5D revealed that only *Il12b*, *Icosl* and *Mki67* gene expression showed statistically significant differences between these two groups (M1-Ita vs. M1), in agreement with the proximity of both groups observed in Figure 1B. It appears that the differential expression of these genes is linked to the loss of the ability to control parasite replication in activated macrophages.

Table 2 summarizes the differential gene expression profiles observed in our experiments. Classically activated macrophages’ antiparasitic activity (M1 vs. Li) depends on activation with IFNG, which induces the enrichment of genes and pathways related to the innate immune response. The pre-stimulus with the M1-associated metabolite itaconate on activated macrophages caused the macrophages to lose their ability to control parasite replication, although the general expression profile was not largely changed (M1-Ita vs. Li) and the enrichment of genes and pathways related to the innate immune and interferon gamma response was maintained. Finally, the particular effect of itaconate, that is, the “conditioning” effect of itaconate pre-stimulus before IFNG activation and infection, was observed in the comparison of M1-Ita and M1: once the activation due to IFNG is eliminated from the equation, the downregulation of *Il12b* and upregulation of both *Icosl* and *Mki67* arise as the only observed effects of itaconate on activated macrophages.

## 4. Discussion

Macrophage activation is a central milestone in *Leishmania* infection, given their important role in host defense as the innate immune response to the infection. In this study, we decided to investigate the transcriptional changes in the macrophage polarization or activation during *L. infantum* infection and the response to metabolic pre-stimulus with itaconic acid. Our approach in the analysis of gene expression changes, which comprised differentially expressed genes (DEGs) associated with a multivariate statistical analysis, led to our unravelling of the most characteristic changes when *L. infantum*-infected macrophages are exposed to different stimuli.

Classically activated macrophages (M1) are traditionally described to produce pro-inflammatory cytokines such as IL6, TNF, IL1B, IL12, IL23 and iNOS upon activation via IFNG and/or TLR stimulation in order to produce reactive oxygen species such as nitric oxide (NO) and its derivatives, which creates an inflammatory environment that contributes to pathogen elimination [4,33,34]. In this study, in order to provide a model for the initial interaction between *Leishmania* and their target host cells (macrophages), firstly, the gene expression profile associated with classically activated macrophages in *L. infantum* infection was determined. In our model of in vitro *L. infantum* infection of macrophages, in classically activated macrophages (M1) but also in classically activated macrophages pre-stimulated with itaconic acid (M1-Ita), most of the traditional markers of M1 polarization or their precursors were upregulated, as seen in Table 2. This activation was correlated with parasitic load reduction in classically activated macrophages, as expected, but not in the IFNG-activated + itaconate pre-stimulated cells (M1-Ita), indicating a deleterious effect of this metabolite on their anti-parasitic activity. Intriguingly, the gene expression profile of these two groups was not too dissimilar, as revealed by multivariate statistical analysis (Figure 1). The rationale behind this finding will be discussed below. Itaconate has been described to have immunoregulatory effects on macrophages, and its accumulation may contribute to the inhibition of glycolysis in LPS-activated macrophages, which is required for optimal inflammatory responses [10,35,36]. M1 or classically activated macrophages show a more glycolytic phenotype, in which the tricarboxylic acid (TCA) cycle is blocked, and this drives the accumulation of citrate, succinate and itaconate (reviewed in [37]). Conversely, M2 macrophages have increased mitochondrial respiration. The anti-inflammatory function of itaconate is associated with succinate dehydrogenase (SDH) inhibition. SDH is an enzyme that catalyzes the oxidation of succinate to fumarate in LPS-activated macrophages and supports the metabolic reprogramming that drives a proinflammatory phenotype [38]. As itaconate leads to SDH inhibition, the accumulation of succinate is associated with the limitation of a series of processes such as mitochondrial respiration, ROS production, proinflammatory cytokine release and inflammasome activation [9,39]. Additionally, another anti-inflammatory mechanism associated with itaconate is the degradation of KEAP1 (Kelch-like ECH-associated protein 1, which targets NRF2 for proteasomal degradation), which, in turn, allows NRF2 to translocate to the nucleus, leading the suppression of the macrophage inflammatory response by blocking proinflammatory cytokine gene transcription (*Il1b* and *Il6*) [9,40].

In our results, the pre-stimulation of macrophages with itaconic acid only induced minor gene expression changes, but when macrophages were concomitantly infected with *L. infantum*, upregulation of genes involved in local acute inflammatory response, particularly *Il6*, was observed, indicating that the anti-inflammatory effect of itaconate weakens the control of parasite replication, and this was evident with the increase in parasite load. This is also supported by the statistically significant upregulation of *Arg1* (an M2 marker involved in the generation of polyamines via l-ornithine, characteristic of M2 activation) in M1-Ita (Table S3), and the lack of differential expression of genes important for the control of parasite replication such as *Il12b* in this group. Therefore, our results reveal that itaconate pre-stimulation diverts the activation of macrophages from a parasite-limiting M1 phenotype towards a more parasite-permissive one, in which only local inflammatory responses mediated by *Il6* are activated.

IL12 secretion is one of the hallmarks of classically activated (M1) macrophages, and, in coordination with other inflammatory markers such as TNFα, is responsible for the production of nitrous oxide (NO) and reactive oxygen species (ROS) via iNOS and NOX2 expression (reviewed in [22]). IL12 is a heterodimeric interleukin, conformed by IL12B (IL-12p40) and IL12A (IL-12p35). IL12 is produced by antigen-presenting cells, and macrophages are considered to be the major source of IL12 in *L. major*-infected mice [16]. In *L. major* infection, the macrophages’ ability to produce IL12 is impaired [41,42,43]. In our model, when macrophages were activated by IFNG and infected with *L. infantum*, *Il12b* was upregulated, and this contributed to the reduction in parasitic burden observed in M1. The pre-stimulation with itaconate abrogated the differential expression of *Il12b* in activated macrophages, and, subsequently, the parasitic load in those macrophages increased (M1-Ita). The particular role of *Il12b* in this process was revealed when both infection and IFNG activation are removed from the equation, i.e., the pre-stimulated, activated and infected macrophages are compared to activated and infected macrophages (M1-Ita vs. M1). In classically activated macrophages pre-stimulated with itaconic acid, the downregulation of *Il12b* and the upregulation of *Icosl* and *Mki67* characterized the effect of itaconic acid on activated macrophages. ICOSL (inducible costimulator ligand) is a cell-surface protein expressed constitutively in dendritic cells, macrophages, B cells and a subset of T cells [44,45]. The binding to its receptor (ICOS) provides costimulatory signals for T-cell proliferation and cytokine secretion [45,46]. Moreover, *Mki67* codes for the Ki-67 protein, which is a nuclear protein involved in cellular proliferation [47]. These proliferation signals could be related to the effect of itaconate on macrophages, taking into account the fact that *Icosl* upregulation was observed not only in classically activated macrophages pre-stimulated with itaconic acid but also in non-activated and uninfected macrophages pre-stimulated with itaconic acid. The fact that itaconic acid has been found to promote tumor cell growth and survival in mouse macrophages also supports this hypothesis [48].

We can conclude that the in vitro pre-stimulus of cells with itaconic acid prior to infection with *L. infantum* increases the parasite load due to the overexpression of inflammatory markers such as *Il6* and *Cxcl9*. Regarding the effect of the pre-stimulus with itaconic acid on macrophage activation, although genes involved in the response to interferon gamma were overexpressed, the parasite load was similar to that observed in the control group; therefore, a greater availability of exogenous itaconate affects macrophage activation with the inhibition of *Il12b*, which is important for the control of parasite replication.

This study clarified the role of itaconate in macrophage activation in *L. infantum* infection. We have observed that exogenous itaconate modulates the immune response to infection of activated macrophages. Although some proinflammatory cytokines were upregulated, which is characteristic of the M1 profile, itaconate accumulation inhibits the antiparasitic function of macrophages, causing an increase in the parasite load. Given our results, the immunoregulatory role of itaconate seems clear. Similarly, in one study, β-glucan counteracted tolerance induced in a model of human endotoxemia by inhibiting the expression of immune-responsive gene 1 (IRG1), the enzyme that controls itaconate synthesis [49]. Considering this, in the context of *Leishmania* infection, an investigation of whether the inhibition of a target point of the IRG1-itaconate-SDH axis can unchain the control of parasite replication is encouraged.

In the last decade, growing evidence has been mounting on the ability of *Leishmania* parasites to manipulate host cells’ metabolic processes in order to mitigate their antiparasitic response (reviewed in [17,22,23,50]). These studies reveal the close interconnection between these intracellular parasites and their host cells, in which metabolic changes at the cellular level determine the polarization towards a more parasite-permissive phenotype. Therefore, the identification of the molecular mechanisms underlying infection may be useful in the search for immunometabolic strategies for infection control in the hosts.

## Figures and Tables

**Table 1:** Experimental groups.

Group	Description	
M0	BMDMs	Non-activated and uninfected macrophages
Li	BMDMs + *L. infantum*	Infected macrophages
M1	BMDMs + IFNG + *L. infantum*	Classically activated macrophages
Li-Ita	BMDMs + itaconic acid + *L. infantum*	Non-activated macrophages + itaconic acid
M1-Ita	BMDMs + itaconic acid + IFNG+ *L. infantum*	Classically activated macrophages + itaconic acid

**Table 2:** Comparative analysis of genes that explained differences in the expression profile of different infected groups. Log_2_FC >1 or Log_2_FC <−1 is indicated in each box; colored boxes represent upregulation (orange) or downregulation (blue). Each group was compared to its corresponding control. FC of genes with *p* ≤ 0.05 between groups are represented in bold.

Gene	M1 (BMDM + *L. infantum* + IFNG) vs. Li (BMDM + *L. infantum*)	M1-Ita (BMDM + *L. infantum* + IFNG + Itaconic Acid) vs. Li (BMDM + *L. infantum*)	M1-Ita (BMDM + *L. infantum* + IFNG + Itaconic Acid) vs. M1 (BMDM + *L. infantum* + IFNG)
*Il12b*	**20.00**		**−20.01**
*Il6*	**21.24**	**20.31**	
*Nos2*	**5.40**	**4.96**	
*Cxcl9*	**21.55**	20.76	
*Il12rb1*	**1.41**	1.55	
*Acod1* (*Irg1*)	**2.99**	**2.74**	
*Stat1*	**1.97**	**2.52**	
*Irf1*	**1.54**	**1.87**	
*Icosl*	−1.45		**1.13**
*Mki67*	**−4.80**	**−3.24**	**1.56**

## Data Availability

Data used in this study are available under request to corresponding author.

## Supplementary Materials

The following supporting information can be downloaded at: https://www.mdpi.com/article/10.3390/tropicalmed8050264/s1, Table S1: List of TaqMan assays used for RT-qPCR analysis using Quant Studio^TM^ 12K Flex Real-Time PCR System; Table S2: Upregulated and downregulated genes in classically activated macrophages (M1) vs. infected macrophages (Li); Table S3: Upregulated and downregulated genes in classically activated macrophages pre-stimulated with itaconic acid (M1-Ita) vs. infected macrophages (Li).
