# Supplementary material for: Gene Expression Profiling of Classically Activated Macrophages in Leishmania infantum Infection: Response to Metabolic Pre-Stimulus with Itaconic Acid
Source: Trop Med Infect Dis. 2023 May 3;8(5):264. doi: 10.3390/tropicalmed8050264 (PMC10223644; doi:10.3390/tropicalmed8050264)
Supplement: Supplementary file 1 [file tropicalmed-08-00264-s001.zip › tropicalmed-2343826-supplementary.pdf]

Tropical Medicine and Infectious Disease

Table S1. List of TaqMan assays used for RT-qPCR analysis using Quant Studio ™ 12K Flex Real-Time PCR System. PANEL 1.

N°	Assay ID	GENE	GENE NAME	GROUP
1	Mm00437762_m1	B2m	beta-2 microglobulin	Endogenous gene expression
2	Mm00446968_m1	Hprt	hypoxanthine guanine phosphoribosyl transferase	
3	Mm00435617_m1	Pgk1	phosphoglycerate kinase 1	
4	Mm01277042_m1	Tbp	TATA box binding protein	
5	Mm01201237_m1	Ubc	ubiquitin C	
6	Mm01722325_m1	Ywhaz	tyrosine 3-monooxygenase/tryptophan 5-monooxygenase activation protein, zeta polypeptide	
7	Mm00599890_m1	Ifngr1	interferon gamma receptor 1	Cytokines and cytokine receptors
8	Mm00492626_m1	Ifngr2	interferon gamma receptor 2	
9	Mm01168134_m1	Ifng	interferon gamma	
10	Mm01178820_m1	Tgfb1	transforming growth factor, beta 1	
11	Mm00436955_m1	Tgfb2	transforming growth factor, beta 2	
12	Mm00436964_m1	Tgfbr1	transforming growth factor, beta receptor I	
13	Mm00436977_m1	Tgfbr2	transforming growth factor, beta receptor II	
14	Mm00443258_m1	Tnf	tumor necrosis factor	
15	Mm00441875_m1	Tnfrsf1a	tumor necrosis factor receptor superfamily, member 1a	
16	Mm00441889_m1	Tnfrsf1b	tumor necrosis factor receptor superfamily, member 1b	
17	Mm01288580_m1	Irf1	interferon regulatory factor 1	
18	Mm00496 7_m1	Irf5	interferon regulatory factor 5	
19	Mm00516788_m1	Irf7	interferon regulatory factor 7	
20	Mm01224532_m1	Irg1	immunoresponsive gene 1	
21	Mm00518984_m1	Il23a	interleukin 23, alpha subunit p19	Interleukines and interleukin receptors
22	Mm00434237_m1	Il1r1	interleukin 1 receptor, type I	
23	Mm00439614_m1	Il10	interleukin 10	
24	Mm00434157_m1	Il10rb	interleukin 10 receptor, beta	
25	Mm00434169_m1	Il12a	interleukin 12a	
26	Mm00434174_m1	Il12b	interleukin 12b	
27	Mm00434189_m1	Il12rb1	interleukin 12 receptor, beta 1	
28	Mm00434200_m1	Il12rb2	interleukin 12 receptor, beta 2	
29	Mm00434204_m1	Il13	interleukin 13	
30	Mm00439618_m1	Il17a	interleukin 17A	
31	Mm00521423_m1	Il17f	interleukin 17F	
32	Mm00434214_m1	Il17ra	interleukin 17 receptor A	
33	Mm00434226_m1	Il18	interleukin 18	
34	Mm00456733_m1	Il18bp	interleukin 18 binding protein	
35	Mm00439620_m1	Il1a	interleukin 1 alpha	
36	Mm00434228_m1	Il1b	interleukin 1 beta	

37	Mm00446186_m1	Il1rn	interleukin 1 receptor antagonist	
38	Mm00434256_m1	Il2	interleukin 2	
39	Mm00517640_m1	Il21	interleukin 21	
40	Mm00600317_m1	Il21r	interleukin 21 receptor	
41	Mm01192969_m1	Il22ra2	interleukin 22 receptor, alpha 2	
42	Mm00519943_m1	Il23r	interleukin 23 receptor	
43	Mm00461162_m1	Il27	interleukin 27	
44	Mm00497259_m1	Il27ra	interleukin 27 receptor, alpha	
45	Mm00442885_m1	Il2rg	interleukin 2 receptor, gamma chain	
46	Mm00445259_m1	Il4	interleukin 4	
47	Mm00439646_m1	Il5	interleukin 5	
48	Mm00434284_m1	Il5ra	interleukin 5 receptor, alpha	
49	Mm00446190_m1	Il6	interleukin 6	
50	Mm00439653_m1	Il6ra	interleukin 6 receptor, alpha	
51	Mm00439665_m1	Il6st	interleukin 6 signal transducer	
52	Mm01290062_m1	Csf2	colony stimulating factor 2 (granulocyte-macrophage)	
53	Mm00655745_m1	Csf2rb	colony stimulating factor 2 receptor, beta, low-affinity (granulocyte-macrophage)	
54	Mm00469294_m1	Ebi3	Epstein-Barr virus induced gene 3	
55	Mm00475988_m1	Arg1	arginase, liver	
56	Mm00475162_m1	Foxp3	forkhead box P3	
57	Mm00440338_m1	Myd88	myeloid differentiation primary response gene 88	Enzymes, prostaglandins and adaptor proteins
58	Mm00440502_m1	Nos2	nitric oxide synthase 2, inducible	
59	Mm00479246_m1	Nox4	NADPH oxidase 4	
60	Mm00478374_m1	Ptgs2	prostaglandin-endoperoxide synthase 2	
61	Mm00439531_m1	Stat1	signal transducer and activator of transcription 1	
62	Mm01219775_m1	Stat3	signal transducer and activator of transcription 3	
63	Mm00448890_m1	Stat4	signal transducer and activator of transcription 4	
64	Mm01160477_m1	Stat6	signal transducer and activator of transcription 6	
65	Mm00477633_m1	Bcl6	B cell leukemia/lymphoma 6	
66	Mm00492590_m1	Ido1	indoleamine 2,3-dioxygenase 1	
67	Mm00500554_m1	Mmp12	matrix metallopeptidase 12	
68	Mm00485054_m1	Mmp14	matrix metallopeptidase 14 (membrane-inserted)	
69	Mm00476361_m1	Nfkb1	nuclear factor of kappa light polypeptide gene enhancer in B cells 1, p105	
70	Mm00479807_m1	Nfkb2	nuclear factor of kappa light polypeptide gene enhancer in B cells 2, p49/p100	
71	Mm00450960_m1	Tbx21	T-box 21	
72	Mm00441891_m1	Cd40	CD40 antigen	
73	Mm00441911_m1	Cd40lg	CD40 ligand	

74	Mm00711660_m1	Cd80	CD80 antigen	Costimulatory and cell adhesion molecules
75	Mm00444543_m1	Cd86	CD86 antigen	
76	Mm00486849_m1	Ctla4	cytotoxic T-lymphocyte-associated protein 4	
77	Mm03048248_m1	Cd274	CD274 antigen	
78	Mm00516023_m1	Icam1	intercellular adhesion molecule 1	
79	Mm00494862_m1	Icam2	intercellular adhesion molecule 2	
80	Mm00497600_m1	Icos	inducible T cell co-stimulator	
81	Mm00497237_m1	Icosl	icos ligand	
82	Mm00434513_m1	Itgb2	integrin beta 2	
83	Mm00486868_m1	Cd83	CD83 antigen	
84	Mm00493071_m1	Lag3	lymphocyte-activation gene 3	
85	Mm01285676_m1	Pdcd1	programmed cell death 1	
86	Mm00454540_m1	Havcr2	hepatitis A virus cellular receptor 2	
87	Mm00441242_m1	Ccl2	chemokine (C-C motif) ligand 2	Chemokines and chemokine receptors
88	Mm00441258_m1	Ccl3	chemokine (C-C motif) ligand 3	
89	Mm00443111_m1	Ccl4	chemokine (C-C motif) ligand 4	
90	Mm01302427_m1	Ccl5	chemokine (C-C motif) ligand 5	
91	Mm00443113_m1	Ccl7	chemokine (C-C motif) ligand 7	
92	Mm01216147_m1	Ccr1	chemokine (C-C motif) receptor 1	
93	Mm01216173_m1	Ccr2	chemokine (C-C motif) receptor 2	
94	Mm01216171_m1	Ccr5	chemokine (C-C motif) receptor 5	
95	Mm01301785_m1	Ccr7	chemokine (C-C motif) receptor 7	
96	Mm04207460_m1	Cxcl1	chemokine (C-X-C motif) ligand 1	
97	Mm00445235_m1	Cxcl10	chemokine (C-X-C motif) ligand 10	
98	Mm00436450_m1	Cxcl2	chemokine (C-X-C motif) ligand 2	
99	Mm00434946_m1	Cxcl9	chemokine (C-X-C motif) ligand 9	
100	Mm00438258_m1	Cxcr2	chemokine (C-X-C motif) receptor 2	
101	Mm00438259_m1	Cxcr3	chemokine (C-X-C motif) receptor 3	
102	Mm00434772_m1	Xcl1	chemokine (C motif) ligand 1	
103	Mm00441260_m1	Ccl9	chemokine (C-C motif) ligand 9	
104	Mm00444533_m1	Cxcl13	chemokine (C-X-C motif) ligand 13	
105	Mm00469712_m1	Cxcl16	chemokine (C-X-C motif) ligand 16	
106	Mm01701838_m1	Cxcl3	chemokine (C-X-C motif) ligand 3	
107	Mm00432086_m1	Cxcr5	chemokine (C-X-C motif) receptor 5	
108	Mm00442346_m1	Tlr2	toll-like receptor 2	Toll-like receptors
109	Mm01207404_m1	Tlr3	toll-like receptor 3	
110	Mm00445273_m1	Tlr4	toll-like receptor 4	
111	Mm00446590_m1	Tlr7	toll-like receptor 7	
112	Mm00446193_m1	Tlr9	toll-like receptor 9	

PANEL 2.

N°	Assay ID	GENE	GENE NAME	GROUP
1	Mm00437762_m1	B2m	beta-2 microglobulin	Endogenous gene expression
2	Mm00446968_m1	Hprt	hypoxanthine guanine phosphoribosyl transferase	
3	Mm01201237_m1	Ubc	ubiquitin C	
4	Mm01331626_m1	Akt1	thymoma viral proto-oncogene 1	MAPK signaling pathway
5	Mm01173094_m1	Akt2	thymoma viral proto-oncogene 2	
6	Mm00442194_m1	Akt3	thymoma viral proto-oncogene 3	
7	Mm01973540_g1	Mapk3, Erk1	mitogen-activated protein kinase 3	
8	Mm00442479_m1	Mapk1, Erk2	mitogen-activated protein kinase 1	
9	Mm00489514_m1	Mapk8, Jnk	mitogen-activated protein kinase 8	
10	Mm00444968_m1	Mtor	mechanistic target of rapamycin (serine/threonine kinase)	
11	Mm01301009_m1	Mapk14, P38mapk	mitogen-activated protein kinase 14	
12	Mm01282781_m1	Pik3r1, Pi3k	phosphatidylinositol 3-kinase, regulatory subunit, polypeptide 1 (p85 alpha)	
13	Mm00440940_m1	Pparg	peroxisome proliferator activated receptor gamma	Lipid metabolism
14	Mm00443451_m1	Nr1h3	nuclear receptor subfamily 1, group H, member 3	
15	Mm00447040_m1	Pla2g4a	phospholipase A2, group IVA (cytosolic, calcium-dependent)	
16	Mm00447271_m1	Ptgis	prostaglandin I2 (prostacyclin) synthase	
17	Mm00441185_m1	Rxra	retinoid X receptor alpha	
18	Mm00436051_m1	Ptger2, Ep	prostaglandin E receptor 2 (subtype EP2)	Protaglandine synthesis
19	Mm00436053_m1	Ptger4	prostaglandin E receptor 4 (subtype EP4)	
20	Mm00452105_m1	Ptges	prostaglandin E synthase	
21	Mm00460181_m1	Ptges2	prostaglandin E synthase 2(Ptges2)	
22	Mm01731378_g1	Ptges3	prostaglandin E synthase 3 (cytosolic)	
23	Mm00477214_m1	Ptgs1	prostaglandin-endoperoxide synthase 1	
24	Mm01199500_m1	P2rx7	purinergic receptor P2X, ligand-gated ion channel, 7	
25	Mm00435472_m1	P2ry2	purinergic receptor P2Y, G-protein coupled 2	
26	Mm00436055_m1	Ptgfr	prostaglandin F receptor	
27	Mm00479846_m1	Hpgds, Ptgds2	intercellular adhesion molecule 1	
28	Mm00482476_m1	Ptgr1, Ltb4dh	prostaglandin reductase 1	
29	Mm00521839_m1	Ltb4r1, Blt1	leukotriene B4 receptor 1	
30	Mm01182747_m1	Alox5	arachidonate 5-lipoxygenase	
31	Mm00507789_m1	Alox15	arachidonate 15-lipoxygenase	
32	Mm00521826_m1	Lta4h	leukotriene A4 hydrolase	

33	Mm00545833_m1	Alox12	arachidonate 12-lipoxygenase	
34	Mm00839636_g1	Cd68	CD68 antigen	Cholesterol pathway
35	Mm00432403_m1	Cd36	CD36 antigen	
36	Mm00459972_m1	Cd209d	CD209d antigen	C-Type Lectin Receptors (CLRs)
37	Mm01183349_m1	Clec7a	C-type lectin domain family 7, member a	
38	Mm01329362_m1	Mrc1	mannose receptor, C type 1	
39	Mm01183378_m1	Cd69	CD69 antigen	
40	Mm00495182_m1	Klrd1, Cd94	killer cell lectin-like receptor, subfamily D, member 1	
41	Mm00435587_m1	Pfkl	phosphofructokinase, liver, B-type	Carbohidrates synthesis
42	Mm00439344_m1	Hk1	hexokinase 2	
43	Mm00443385_m1	Hk2	hematopoietic prostaglandin D synthase	
44	Mm00441480_m1	Slc2a1	solute carrier family 2 (facilitated glucose transporter), member 1	
45	Mm00472712_m1	Gys1	granzyme B	
46	Mm00504650_m1	Pfkfb3	6-phosphofructo-2-kinase/fructose-2,6-biphosphatase 3	
47	Mm00554300_m1	Pdk1	pyruvate dehydrogenase kinase, isoenzyme 1	
48	Mm01268229_m1	Pgm2	phosphoglucomutase 2	
49	Mm01612132_g1	Ldha	lactate dehydrogenase A	
50	Mm00833691_g1	Tpi1	triosephosphate isomerase 1	
51	Mm00434151_m1	Il10ra	interleukin 10 receptor, beta	Interleukines and interleukin receptors
52	Mm00446726_m1	Il13ra1	interleukin 13 receptor, alpha 2	
53	Mm00515166_m1	Il13ra2	interleukin 15	
54	Mm00434210_m1	Il15	interleukin 16	
55	Mm00516039_m1	Il16	interleukin 17 receptor A	
56	Mm00515178_m1	Il18r1	interleukin 18 receptor accessory protein	
57	Mm00516053_m1	Il18rap	interleukin 1 alpha	
58	Mm00663697_m1	Il22ra1	interleukin 23, alpha subunit p19	
59	Mm01340213_m1	Il27ra	interleukin 2 receptor, alpha chain	
60	Mm00434268_m1	Il2rb	interleukin 2 receptor, gamma chain	
61	Mm01275139_m1	Il4ra	interleukin 5 receptor, alpha	
62	Mm00434295_m1	Il7r, Cd127	interleukin 9 receptor	
63	Mm00434313_m1	Il9r	interferon regulatory factor 1	
64	Mm00499822_m1	Il25	interleukin 25	
65	Mm00434305_m1	Il9	interleukin 9	
66	Mm00499822_m1	Il2ra, Cd25	interleukin 2 receptor, alpha chain	
67	Mm00444241_m1	Il22	interleukin 22	
68	Mm00516136_m1	Ccl17	chemokine (C-C motif) ligand 17	Chemokines and chemokine receptors
69	Mm00839966_g1	Ccl19	chemokine (C-C motif) ligand 19	
70	Mm00436439_m1	Ccl22	chemokine (C-C motif) ligand 22	
71	Mm00438271_m1	Ccr4	chemokine (C-C motif) receptor 4	

72	Mm00444662_m1	Cxcl11	chemokine (C-X-C motif) ligand 11	
73	Mm01292123_m1	Cxcr4	chemokine (C-X-C motif) receptor 4	
75	Mm00472858_m1	Cxcr6	Epstein-Barr virus induced gene 3	
76	Mm00441263_m1	Cxcl15	chemokine (C-X-C motif) ligand 15	
77	Mm00490880_m1	Stat2	signal transducer and activator of transcription 2	Enzymes and adaptor proteins
78	Mm00600614_m1	Jak1	Janus kinase 1	
79	Mm01208489_m1	Jak2	Janus kinase 2	
80	Mm00477631_m1	Bcl2	B cell leukemia/lymphoma 2	
81	Mm00432050_m1	Bax	BCL2-associated X protein	
82	Mm00437783_m1	Bcl2l1	BCL2-like 1	
83	Mm00438861_m1	Fadd	Fas (TNF receptor superfamily member 6)	
84	Mm00442834_m1	Gzmb	hepatitis A virus cellular receptor 2	
85	Mm00436979_m1	Tgm2	transglutaminase 2, C polypeptide	
86	Mm00445109_m1	Retnla, Fizz1	resistin like alpha	
87	Mm00448427_m1	Ptpn1, Ptp1b	protein tyrosine phosphatase, non-receptor type 1	
88	Mm01278617_m1	Mki67, Ki67	antigen identified by monoclonal antibody Ki 67	
89	Mm00661498_m1	CD57, B3gat1	beta-1,3-glucuronyltransferase 1 (glucuronosyltransferase P)	
90	Mm00451734_m1	Pdcd1lg2	programmed cell death 1 ligand 2	Costimulatory and cell adhesion molecules
91	Mm00599683_m1	Cd3e	CD3 antigen, epsilon polypeptide	
92	Mm01182108_m1	Cd8a	CD8 antigen, alpha chain	
93	Mm00488332_m1	Siglec1, Cd169	sialic acid binding Ig-like lectin 1, sialoadhesin	
94	Mm00434455_m1	Itgam	integrin alpha M	
95	Mm00483137_m1	Cd28	CD28 antigen	
96	Mm00801807_m1	Itgal	integrin alpha M	
97	Mm00444461_m1	Cd160	CD160 antigen	
98	Mm01251919_m1	Itgae, Cd103	integrin alpha L	
99	Mm00442754_m1	Cd4	CD4 antigen(Cd4)	
100	Mm01149710_m1	Ncam1, Cd56	neural cell adhesion molecule 1	
101	Mm00484683_m1	Gata3	glycogen synthase 1, muscle	Transcription factors
102	Mm00467257_m1	Nfat5	nuclear factor of activated T cells 5	
103	Mm00468869_m1	Hif1a	hexokinase 1	
104	Mm00627599_m1	Ucp2	uncoupling protein 2 (mitochondrial, proton carrier)	Carrier proteins
105	Mm00437136_m1	Tnfrsf18	tumor necrosis factor receptor superfamily, member 18	Cytokines and cytokine receptors
106	Mm00616981_m1	Btla	B and T lymphocyte associated	

107	Mm00438864_m1	Fasl	forkhead box P3	
108	Mm01204974_m1	Fas	Fas ligand (TNF superfamily, member 6)	
109	Mm00442039_m1	Tnfrsf4	tumor necrosis factor receptor superfamily, member 4	
110	Mm00440228_gH	Lta	lymphotoxin A	
111	Mm01342740_g1	Socs1	suppressor of cytokine signaling 1	
112	Mm01249143_g1	Socs3	suppressor of cytokine signaling 3	

Table S2. Upregulated and downregulated genes in classically activated macrophages (M1) vs. infected macrophages (Li). List of DEGs or genes with factor Loading PC1 ≥ 0,7 considered as upregulated in classically activated macrophages (M1) vs. infected macrophages (Li) and list of DEGs or genes with factor Loading PC1 ≤ -0,7 considered as downregulated. Genes are ordered by Log_2FC. DEGs are in bold.

**UPREGULATED** (DEGs or Factor Loading PC1 ≥ 0,7)	Log2(FC)	*p*-value	Factor loading
Cxcl9	21,55	0,04	-0,99
Il6	21,24	0,05	-0,95
Cxcl11	20,73	0,04	-0,89
Il12b	20,01	0,04	-0,88
Arg1	19,53	0,05	-0,84
Pdcd1lg2	19,35	0,05	-0,82
Ccl22	18,93	0,05	-0,72
Nos2	5,39	0,05	-0,96
Ptges3	3,97	0,12	-0,71
Acod1	3,00	0,05	-0,99
Il18bp	2,54	0,05	-0,99
Stat1	1,97	0,05	-0,98
Cd274	1,88	0,05	-0,94
Irf1	1,54	0,05	-0,96
Il12rb1	1,41	0,08	-0,92
Icam2	1,36	0,08	-0,74
Ptges	1,35	0,05	-0,89
Cxcl2	1,27	0,12	-0,78
Ccl5	1,21	0,08	-0,70
Ccr1	1,10	0,12	-0,78
Tgm2	0,89	0,08	-0,96
Cd40	0,86	0,05	-0,71
Il27	0,85	0,12	-0,82
Mmp12	0,70	0,05	-0,88
Il2rg	0,65	0,05	-0,86
Irf7	0,60	0,05	-0,75
Tlr9	0,28	0,13	-0,73

**DOWNREGULATED** (DEGs or Factor loading PC1 ≤ -0,7)	Log2(FC)	*p*-value	Factor loading
Il12rb2	-19,97	0,05	0,81
Ptgis	-19,50	0,05	0,85
Mki67	-4,80	0,05	0,97
Cd28	-3,58	0,05	0,99
Alox5	-3,52	0,05	0,97
Mrc1	-3,40	0,08	0,81
Stat4	-3,35	0,08	0,90
Tgfb2	-3,24	0,05	0,99
Il10	-3,23	0,12	0,72
Ccl9	-2,67	0,05	0,98
Cd4	-2,61	0,05	0,98
Ifngr1	-2,47	0,08	0,94
Ptgs1	-2,32	0,08	0,92
Cxcl13	-2,19	0,12	0,90
Mapk3	-2,15	0,05	0,95
Akt1	-2,13	0,08	0,92
Il10ra	-2,06	0,12	0,88
Nox4	-1,88	0,08	0,83
Ebi3	-1,80	0,12	0,75
Cd68	-1,79	0,05	0,89
Il1r1	-1,78	0,08	0,83
Cxcr4	-1,76	0,05	0,98
Hif1a	-1,72	0,05	0,99
Fadd	-1,70	0,12	0,83
Ifngr2	-1,66	0,05	0,90
Pik3r1	-1,55	0,08	0,82
Cxcr3	-1,54	0,12	0,85
Il16	-1,52	0,05	0,88
Mapk1	-1,46	0,12	0,84
Il17ra	-1,41	0,08	0,74
Il1b	-1,41	0,05	0,96
Siglec1	-1,35	0,08	0,79

Tlr2	-1,32	0,05	0,95
Hk2	-1,28	0,08	0,81
Il6ra	-1,26	0,05	0,92
P2rx7	-1,25	0,05	0,89
Rxra	-1,24	0,05	0,91
Hpgds	-1,23	0,05	0,94
Mmp14	-1,19	0,05	0,98
Lta4h	-1,17	0,05	0,91
Cd83	-1,16	0,08	0,83
Tgfbr1	-1,13	0,08	0,85
Il1rn	-1,11	0,05	0,90
Tgfbr2	-1,10	0,05	0,99
Il6st	-1,09	0,05	0,90
Ucp2	-1,07	0,08	0,87
Bcl6	-1,00	0,08	0,72
Ptger4	-1,00	0,08	0,91
Bax	-0,99	0,08	0,80
Gys1	-0,98	0,13	0,79
Akt3	-0,94	0,05	0,90
Nfat5	-0,90	0,05	0,86
Tgfb1	-0,89	0,05	0,98
Ncam1	-0,89	0,08	0,72
Ptgr1	-0,84	0,05	0,82
Nfkb2	-0,80	0,05	0,91
Il18	-0,76	0,08	0,71
Mapk14	-0,72	0,05	0,89
Myd88	-0,71	0,05	0,95
Il13ra1	-0,70	0,08	0,86
Tlr7	-0,68	0,08	0,87
Tlr4	-0,67	0,05	0,89
Hk1	-0,67	0,08	0,78
Nfkb1	-0,66	0,05	0,99
Itgal	-0,62	0,05	0,81
Tnfrsf1a	-0,60	0,05	0,91
Itgam	-0,57	0,05	0,82
Il4ra	-0,54	0,05	0,76
Jak1	-0,53	0,08	0,88
Il1rap	-0,52	0,13	0,82
Ldha	-0,51	0,13	0,72
Pfkl	-0,28	0,08	0,72

Table S3. Upregulated and downregulated genes in classically activated macrophages pre-stimulated with itaconic acid (M1-Ita) vs. infected macrophages (Li). List of DEGs or genes with factor Loading PC1 ≥ 0,7 considered as upregulated in classically activated macrophages pre-stimulated with itaconic acid (M1-Ita) vs. infected macrophages (Li) and list of DEGs or genes with factor Loading PC1 ≤ -0,7 considered as downregulated. Genes are ordered by Log_2FC. DEGs are in bold.

**UPREGULATED** (DEGs or Factor Loading PC1 ≥ 0,7)	Log2(FC)	*p*-value	Factor loading
Arg1	21,46	0,04	-0,83
Cxcl9	20,76	0,05	-0,83
Il6	20,31	0,04	-0,96
Cxcl11	19,37	0,05	-0,75
Nos2	4,96	0,05	-0,98
Ptges3	3,14	0,08	-0,96
Acod1	2,74	0,05	-0,98
Stat1	2,52	0,05	-0,98
Ccr1	2,18	0,12	-0,81
Icam2	2,01	0,12	-0,79
Irf1	1,87	0,05	-0,97
Ptges	1,52	0,05	-0,91
Il27	1,50	0,08	-0,94
Tgm2	1,03	0,08	-0,94
Il2rg	1,02	0,05	-0,95
Tnfrsf1b	0,88	0,05	-0,95
Irf7	0,84	0,05	-0,89
Il1a	0,34	0,13	-0,74

**DOWNREGULATED** (DEGs or Factor loading PC1 ≤ -0,7)	Log2(FC)	*p*-value	Factor loading
Nox4	-20,46	0,10	0,85
Il6ra	-20,10	0,08	0,84
Il12rb2	-19,97	0,05	0,80
Ptgis	-19,50	0,05	0,84
Stat4	-3,91	0,08	0,91
Mki67	-3,24	0,05	0,96
Cd28	-3,06	0,05	0,98
Mrc1	-2,96	0,08	0,80
Tgfb2	-2,72	0,05	0,98
Ccl9	-2,65	0,08	0,97
Cd4	-2,48	0,08	0,93
Cd80	-2,37	0,05	0,68
Akt1	-2,33	0,12	0,89
Fadd	-2,08	0,08	0,83
Ifngr1	-2,05	0,08	0,92
Il10ra	-2,01	0,12	0,87
Alox5	-1,92	0,05	0,93
Il1r1	-1,91	0,08	0,84
Ifngr2	-1,65	0,08	0,81
Ptgs1	-1,63	0,05	0,89
Bax	-1,61	0,12	0,80
Cd83	-1,59	0,12	0,81
Mapk1	-1,54	0,12	0,89
Siglec1	-1,44	0,12	0,72
Il1rn	-1,38	0,05	0,92
Mapk3	-1,35	0,05	0,91
Ncam1	-1,31	0,08	0,76
Cxcr4	-1,25	0,08	0,97
Il16	-1,20	0,05	0,83
Il1b	-1,19	0,05	0,96
Cd68	-1,13	0,13	0,80
Ptgfr	-1,07	0,13	0,71

Jak1	-1,01	0,08	0,87
Mapk8	-1,00	0,08	0,86
Il6st	-0,96	0,05	0,90
Hpgds	-0,95	0,05	0,96
Ptger4	-0,94	0,05	0,92
Hif1a	-0,93	0,08	0,97
Nfat5	-0,82	0,05	0,86
Akt3	-0,77	0,05	0,80
Pik3r1	-0,73	0,13	0,77
Rxra	-0,72	0,05	0,83
Nfkb2	-0,67	0,05	0,95
P2rx7	-0,58	0,13	0,75
Tgfb1	-0,56	0,05	0,96
Tlr2	-0,54	0,05	0,87
Hk1	-0,50	0,08	0,72
Mmp14	-0,49	0,05	0,89
Lta4h	-0,44	0,13	0,70
Tgfbr1	-0,43	0,05	0,88
Pdk1	-0,38	0,13	0,74
Nfkb1	-0,35	0,05	0,87
Mapk14	-0,30	0,08	0,75
Tnfrsf1a	-0,28	0,05	0,95
